# ncPred: ncRNA-Disease Association Prediction through Tripartite Network-Based Inference

**DOI:** 10.3389/fbioe.2014.00071

**Published:** 2014-12-12

**Authors:** Salvatore Alaimo, Rosalba Giugno, Alfredo Pulvirenti

**Affiliations:** ^1^Department of Mathematics and Computer Science, University of Catania, Catania, Italy; ^2^Department of Clinical and Experimental Medicine, University of Catania, Catania, Italy

**Keywords:** ncRNAs-diseases association predictions, lncRNAs functional characterization, network-based inference, tripartite networks, resource transfer algorithm

## Abstract

**Motivation:** Over the past few years, experimental evidence has highlighted the role of microRNAs to human diseases. miRNAs are critical for the regulation of cellular processes, and, therefore, their aberration can be among the triggering causes of pathological phenomena. They are just one member of the large class of non-coding RNAs, which include transcribed ultra-conserved regions (T-UCRs), small nucleolar RNAs (snoRNAs), PIWI-interacting RNAs (piRNAs), large intergenic non-coding RNAs (lincRNAs) and, the heterogeneous group of long non-coding RNAs (lncRNAs). Their associations with diseases are few in number, and their reliability is questionable. In literature, there is only one recent method proposed by Yang et al. (2014) to predict lncRNA-disease associations. This technique, however, lacks in prediction quality. All these elements entail the need to investigate new bioinformatics tools for the prediction of high quality ncRNA-disease associations. Here, we propose a method called *ncPred* for the inference of novel ncRNA-disease association based on recommendation technique. We represent our knowledge through a tripartite network, whose nodes are ncRNAs, targets, or diseases. Interactions in such a network associate each ncRNA with a disease through its targets. Our algorithm, starting from such a network, computes weights between each ncRNA-disease pair using a multi-level resource transfer technique that at each step takes into account the resource transferred in the previous one.

**Results:** The results of our experimental analysis show that our approach is able to predict more biologically significant associations with respect to those obtained by Yang et al. (2014), yielding an improvement in terms of the average area under the ROC curve (AUC). These results prove the ability of our approach to predict biologically significant associations, which could lead to a better understanding of the molecular processes involved in complex diseases.

**Availability:** All the *ncPred* predictions together with the datasets used for the analysis are available at the following url: http://alpha.dmi.unict.it/ncPred/

## 1 Introduction

In recent years, great efforts have been employed in the study of non-coding RNAs (ncRNAs), a class of genes involved in a wide variety of biological functions. Small ncRNAs, such as siRNA, miRNA, and piRNA, are highly conserved in different species and have a key role in transcriptional and post-transcriptional silencing of genes. Long ncRNA (transcribed RNA molecules whose length is greater than 200 nucleotides) instead are poorly preserved and have the task of regulating gene expression through mechanisms still largely unknown (Mercer et al., 2009; Ponting et al., 2009; Wilusz et al., 2009). It has been shown that these molecules are involved in the regulation of gene expression by acting as controllers of processes such as RNA maturation or transportation, or altering chromatin structure. ncRNAs have great variety in structure and in gene regulation outcomes, however, several similarities can be identified in the way they act (Wang and Chang, 2011).

The connection between diseases and de-regulation of small ncRNAs has been established for years. However, recent studies show that mutations and de-regulations of lncRNAs are heavily involved in the development or progression of several diseases (Wapinski and Chang, 2011). Alterations in the structure (primary or secondary), or in the expression levels are the main underlying causes of diseases, from cancer to neurodegenerative disorders (Wapinski and Chang, 2011).

Pasmant et al. (2011) highlight how the expression of the lncRNA *ANRIL*, antisense transcript to *INK4b* gene, is correlated with the epigenetic silencing of *INK4a*, or *p16 protein*, which is involved in the regulation of cell cycle. High levels of *ANRIL* were found in prostate cancer tissues (Yap et al., 2010). Yap et al. (2010), also, hypothesizes that this transcript is an initiating factor in tumor formation due to its silencing action on the *INK4b/ARF/INK4a* locus. Other experimental evidence link *ANRIL* de-regulation to a number of pathologies, including coronary disease, intracranial aneurysm, and type II diabetes (Pasmant et al., 2011).

Another example of correlation between lncRNAs and diseases is the *HOTAIR* transcript, which is involved in the progression of breast cancer by chromatin landscape remodeling (Burd et al., 2010). In particular, increased expression of tHOTAIR is an index of poor prognosis and tumor metastasis. Gupta et al. (2010) show that *HOTAIR* is also responsible for invasiveness and metastasis in epithelial cancer cells and its inhibition may lead to a reduction of invasiveness in cells where *PRC2 complex* is highly activated.

Further evidence of lncRNAs-diseases correlation is the transcript called *MALAT-1*, an RNA of more than 8000*nt* present in chromosome 11*q*13, whose over-expression is related to bad prognosis in patients with non-small cell lung cancer (Ji et al., 2003). In addition, the antisense transcript of *β-secretase-1* (*BACE1-AS*) has been identified in high concentrations in subjects with Alzheimer’s disease and in amyloid precursor protein transgenic mice (Faghihi et al., 2008).

Therefore, despite the enormous importance that ncRNAs show in connection with several diseases, the number of entities, which somehow has been functionally characterized and associated to diseases, is extremely small (Wapinski and Chang, 2011). For this purpose, the developing a methodology that is able to predict ncRNA-disease interactions is crucial in order to formulate new hypotheses on the molecular mechanisms underlying complex diseases, and to identify potential new biomarkers for their diagnosis, treatment and prevention. Despite the use of such a methodology could be very helpful by making the search for new associations more focused and less costly, it must be emphasized that the task of determining, which are beneficial remains a responsibility of bio-physicians. They, indeed by identifying appropriate patient groups and properly documenting such cases, can establish the actual relationship, while also allowing a broader understanding of the underlying phenomena.

In this direction, Yang et al. (2014) developed a method, which exploits a bipartite network and a propagation algorithm to predict new associations that can be evaluated through appropriate *in vitro* experiments. Yang et al. (2014) based their method on the database assembled by Chen et al. (2013): a collection of approximately 1028 experimentally validated interactions among 322 lncRNAs and 221 diseases. The database has been further extended, through deep literature mining, to include additional interactions. The database includes also 478 experimentally validated interactions among 126 lncRNAs and 236 protein coding genes. For such genes a modulation in expression values is known to be carried out by such ncRNAs.

In this paper we present *ncPred*, a resource propagation methodology, which uses a tripartite network to guide the inference process of novel ncRNA-disease associations. The tripartite network allows the introduction of two levels of interaction: ncRNA-target and target-disease. Here, we call targets a group of biomolecules (i.e., genes, microRNAs, proteins) whose activity is modulated by a ncRNA (e.g., regulation of expression, binding to improve the efficiency of its activity, or binding to help the formation of complexes). In this way, we can exploit the greater quantity of known interactions between targets (i.e., proteins and miRNAs) and diseases to build a wider knowledge base and obtain a greater number of high quality predictions.

To perform a proper evaluation of our method, we applied a k-fold Cross-Validation procedure to the (Chen et al., 2013) database, remodeled to include information on targets. A further analysis uses a database of experimentally verified interactions between ncRNAs and miRNAs shown in Helwak et al. (2013).

## 2 Materials and Methods

### 2.1 Algorithm

Let *O* = {*o*_1_, *o*_2_, …, *o_n_*} be a set of non-coding RNAs (ncRNAs), let *T* = {*t*_1_, *t*_2_, …, *t_m_*} be a set of targets (i.e., genes, microRNA), and let *D* = {*d*_1_, *d*_2_, …, *d_p_*} be a set of diseases. The ncRNA-target and target-disease interactions can be represented in a tripartite graph *G*(*O*, *T*, *D*, *E*), where *E* is the set of interactions (edges) between nodes in *O* and *T* and nodes in *T* and *D*. Such a graph, can be represented by using a pair of adjacency matrices $A^{\mathrm{OT}}={\left\{a_{\mathrm{ij}}^{\mathrm{OT}}\right\}}_{n\times m}$ and $A^{\mathrm{TD}}={\left\{a_{\mathrm{rs}}^{\mathrm{TD}}\right\}}_{m\times p}$ where $a_{\mathrm{ij}}^{\mathrm{OD}}=1$ if *o_i_* is connected to *t_j_* in *G*, and $a_{\mathrm{rs}}^{\mathrm{TD}}=1$ if *t_r_* is connected to *d_s_* in *G*.

Our technique is based on the concept of resources transfer within the network. We refer to Alaimo et al. (2013) for details of resources transfer (drug-targeting) in bipartite networks. The bipartite network carries a prior knowledge which can be used to infer novel interactions. Starting from such a network, it computes weights between each pair of target. Those weights can be seen as the likelihood by which we can affirm that if a drug is associated with a target then it may be associated with another one. For each prediction, the algorithm also associates a score indicating the degree of certainty of the interaction.

**Figure 1:**
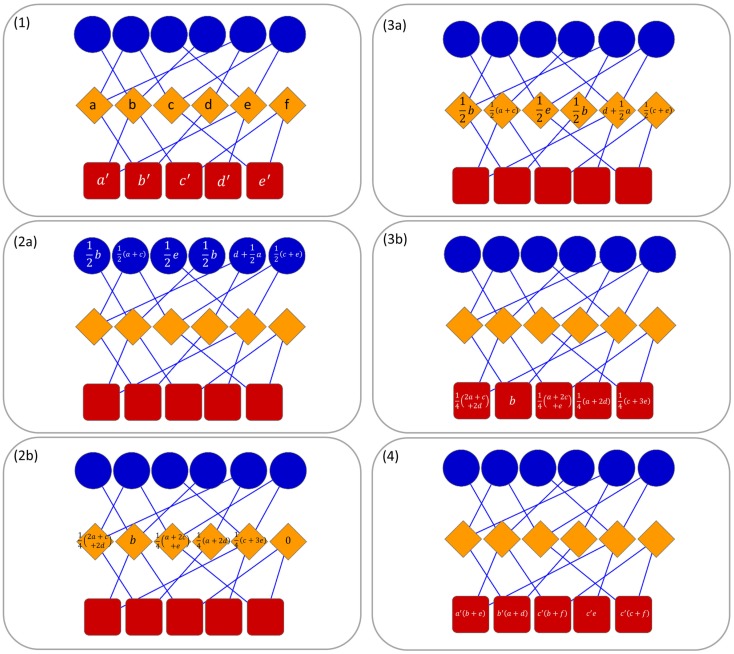
**Operating principle of ncPred in a tripartite network**. Here, we represent ncRNAs in blue, targets in orange, and diseases in red. Without loss of generality, and in order to simplify the reading of the image, we decided to put *λ_1_* and *λ_2_* to 1, so as to obtain a uniform distribution of resources in the network. In the first step, a resource is assigned to each target and disease node (1). Thereafter, two separate transfer process are launched to compute the resource in target nodes (2a, 2b) and disease nodes (3a, 3b). Finally, resources are combined to obtain the total quantity in each disease node (4). In (4), the literals are used only for example purposes due to lack of space. They are to be replaced with the values computed in steps (2b) and (3b).

In this paper, due to the tripartite network, we developed a multi-level transfer approach that at each step takes into account the resource transferred in the previous one (see Figure 1 for an example). In the first level of the transfer, the resource is moved from the nodes in *T* (targets) to nodes in *O* (ncRNAs) and vice versa. In the second level, the resource is moved from *D* nodes to *T* nodes and it is combined with the resource of the previous step. Then, the resources are moved back to the *D* nodes. In this way, we define a methodology for the computation of a combined weight matrix $W^{C}={\left\{w_{\mathrm{ij}}^{c}\right\}}_{m\times p},$ where $w_{\mathrm{ij}}^{c}$ corresponds to the likelihood allowing us to claim that if a ncRNA interacts with a target *t_i_* then it may be associated with the pathology *d_j_*.

To compute such a matrix, we start by defining two partial weight matrices corresponding to the intermediate levels of transfer. These two matrices are then used to obtain the combined weight matrix and, therefore, compute the recommendations.

Let *k*′(*x*) be the degree of node *x* in the ncRNA-target sub-network and *k*″(*y*) the degree of node *y* in the target-disease sub-network.

The matrix $W^{T}={\left\{w_{\mathrm{ij}}^{T}\right\}}_{m\times m},$ associated with the first level of transfer, can be defined as:

(1) $$w_{\mathrm{ij}}^{T}=\frac{1}{k^{\prime }{\left(t_{i}\right)}^{\left(1-\lambda_{1}\right)}k^{\prime }{\left(t_{j}\right)}^{\lambda_{1}}}\sum_{l=1}^{n}\frac{a_{\mathrm{li}}^{\mathrm{OT}}a_{\mathrm{lj}}^{\mathrm{OT}}}{k^{\prime }\left(o_{l}\right)},$$

where $w_{\mathrm{ij}}^{T}$ corresponds to the likelihood that given a ncRNA interacting with target *t_i_*, then it may also interact with target *t_j_*. By using such an equation, we assign higher weights to the pairs of targets that share many ncRNAs, rather than those who share only a few.

The same applies to $W^{D}={\left\{w_{\mathrm{ij}}^{D}\right\}}_{p\times p},$ matrix associated with the second level of the transfer, where:

(2) $$w_{\mathrm{ij}}^{D}=\frac{1}{k^{\prime \prime }{\left(d_{i}\right)}^{\left(1-\lambda_{2}\right)}k^{\prime \prime }{\left(d_{j}\right)}^{\lambda_{2}}}\sum_{l=1}^{m}\frac{a_{\mathrm{li}}^{\mathrm{TD}}a_{\mathrm{lj}}^{\mathrm{TD}}}{k^{\prime \prime }\left(t_{l}\right)}.$$

In equation 2, $w_{\mathrm{ij}}^{D}$ indicates whether we can assert that given a target associated with the disease *d_i_*, it may also be linked to the disease *d_j_*. $w_{\mathrm{ij}}^{D}$ is higher for the disease pairs, which are associated to many common targets with respect to those with fewer common targets.

In equations 1 and 2, the *λ*_1_ ∈ [0, 1] and *λ*_2_ ∈ [0, 1] parameters are used to tune the quality of the predictions. Parameter values close to zero indicate that the resource of a node is computed as the average of those in its neighborhood, while values close to one indicate that the resource is uniformly distributed among the nodes of its neighborhood. In terms of predictions, lambda values close to zero correspond to conservative predictions, while values close to one correspond to a larger number of predictions.

Therefore, the combined weight matrix $W^{C}={\left\{w_{\mathrm{ij}}^{c}\right\}}_{m\times p}$ can be obtained as:

(3) $$w_{\mathrm{ij}}^{C}=\sum_{t=1}^{m}\left[w_{\mathrm{it}}^{T}\sum_{r=1}^{p}\left(a_{\mathrm{tr}}^{\mathrm{TD}}\cdot w_{\mathrm{rj}}^{D}\right)\right].$$

In equation 3, the weight of a target-disease pair is computed by taking into account both the targets with a similar neighborhood and the diseases with a similar neighborhood. In this way, a larger weight is assigned to those pairs for which more frequently there is a path, which passes through them.

Given the above weights, it is now possible to compute the recommendation matrix *R* = {*r_ij_*}*_n_*_×_*_p_* as:

(4) $$R=A^{\mathrm{OT}}\cdot W^{C}.$$

We call each *r_ij_* prediction score for the pair (*i*, *j*). For each ncRNA *o_i_*, its list of predictions *R_i_* can be obtained by selecting those disease-prediction score pairs for which there is no path with *o_i_* in the tripartite network. Such a list is sorted in descending order with respect to the value of *r_ij_*, as the higher the score, the greater the belief that the ncRNA will have some connection with that particular disease.

### 2.2 Datasets and benchmarks

We evaluated our method using two datasets containing experimentally verified interactions between ncRNAs, targets, and diseases. The first data set (Figure S1 in Supplementary Material) was built by collecting from (Chen et al., 2013) 478 interactions between lncRNAs and genes. These interactions were mapped by converting each target identifier to its Entrez Id. This allowed us to remove about 230 duplicates or superseded interactions. From the remaining targets, we then extracted 1005 experimentally validated gene-disease associations by searching in DisGeNET (Bauer-Mehren et al., 2010).

**Figure 2:**
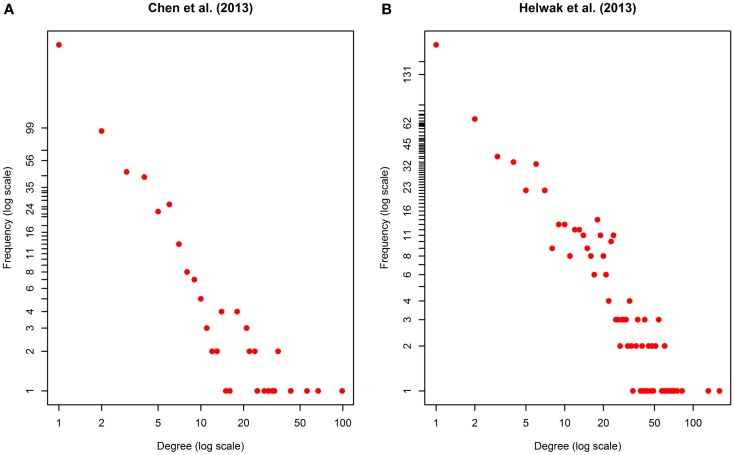
**Degree distribution of the two networks used as datasets**: **(A)** Chen et al. (2013), **(B)** Helwak et al. (2013). The two plots are in log-log scale. As can be seen the degree distribution for the two networks can be approximated to an exponential one. We can therefore assume that the two networks are scale-free.

The second data set (Figure S2 in Supplementary Material) was obtained by collecting about 4000 lncRNA-miRNA interactions found by Helwak et al. (2013) by applying the CLASH methodology (Kudla et al., 2011). Each association indicates that a lncRNA contains one or more binding sites for miRNAs. From such a list, we removed all targets not present in miR2Disease database (Jiang et al., 2009), obtaining 1699 lncRNA-miRNA associations. Finally, using Jiang et al. (2009), we recovered 1572 miRNA-disease associations. Table 1 provides a summary of the two datasets together with some metrics that can further elucidate their characteristics. Moreover, in Figure 2, we calculated the degree distribution of the two networks. These show that they can be considered scale-free networks.

**Table 1:** **Description of the datasets: number of ncRNAs, targets and diseases together with the count of interactions, average degree, density, modularity, number of connected components, and average path length**.

Metrics	Chen et al. (2013)	Helwak et al. (2013)
ncRNAs	119	338
Targets	110	179
Diseases	514	134
ncRNAs–targets interactions	247	1699
Targets–diseases interactions	1005	1572
Average degree	1.572	5.025
Density	0.002	0.008
Modularity	0.609	0.274
Number of connected components	24	1
Average path length	1.572	1.734

For the evaluation of our method, we applied a 10-fold cross-validation procedure repeated 30 times to obtain more reliable results. Each fold is built in the following way. Given the tripartite graph, we selected all possible pairs of ncRNA-disease interactions. Then, we randomly partitioned them into each fold. We make sure that the tripartite network generated from each fold is not disconnected. *ncPred* makes predictions only on connected networks. We considered the following four metrics (Alaimo et al., 2013) to assess the performance of our method: precision and recall enhancement, recovery, personalization, and Surprizal. The first two establish the ability of the method to recover the interactions of the test set, therefore, obtaining biologically relevant predictions. The other two measure the ability of the method to propose unexpected interactions, which may lead to novel insights onto ncRNA functions. Special care should be given to the precision and recall enhancement metrics. They measure the reliability of the prediction algorithm by comparing the standard precision and recall with a null model. Such a model is defined as a methodology that randomly assigns ncRNA-disease pairs. This implies that values greater than one are to be considered synonymous of higher quality and, therefore, reliability.

## 3 Results

As stated earlier, to evaluate the power of our method, we applied a 10-fold cross-validation procedure repeated 30 times and averaged results to obtain more reliable estimates. In Table 2, we illustrate the behavior of *ncPred*, comparing it with Yang et al. (2014), in terms of precision and recall enhancement. The results demonstrate that *ncPred* clearly outperforms its competitor. In particular, we can see that while Yang et al. (2014) obtains a recall close to the null model, *ncPred* has much better results. This is crucial since the recall measures the ability of the algorithm to recover existing interactions in the network, and is therefore a sign of their reliability, namely their biological relevance.

**Table 2:** **Comparison of ncPred and Yang et al. (2014) through the precision and recall enhancement metric, and the average area under ROC curve (AUC) calculated for each of the two datasets listed in Table 1**.

Dataset	*e_P_*(20)		*e_R_*(20)		*AUC*(20)	
	Yang et al. (2014)	ncPred	Yang et al. (2014)	ncPred	Yang et al. (2014)	ncPred
Chen et al. (2013)	5.5113	12.3290	0.7297	1.6636	0.6217 ± 0.0178	0.7566 ± 0.0218
Helwak et al. (2013)	1.8654	5.8197	1.6509	5.6572	0.7069 ± 0.0084	0.7669 ± 0.0093

*The results were obtained using the optimal values for *λ_1_* and *λ_2_* parameters as shown in Table 3*.

**Figure 3:**
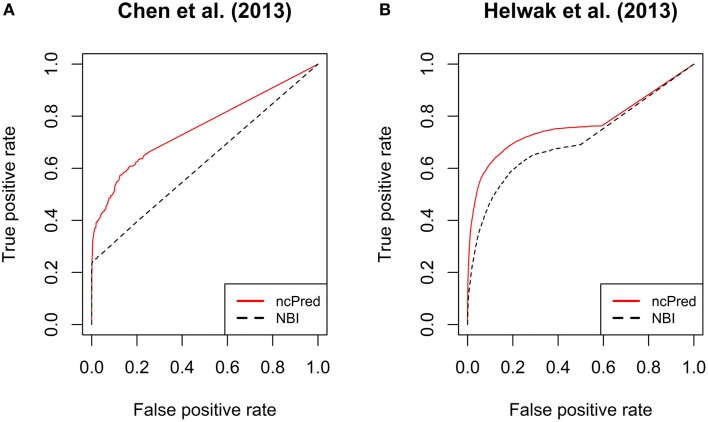
**Comparison between ncPred and Yang et al. (2014) by means of receiver operating characteristic (ROC) curves, computed for the recommendation lists built on our two datasets**. Such curves measure the quality of the algorithms in terms of false positives rate against true positives rate. **(A,B)** are independent since computed on two separate datasets. The significance of the difference highlighted between ncPred and Yang et al. (2014) was measured by applying the Friedman rank sum test as assessed in Table 4.

In Figure 3, we report the receiver operating characteristic (ROC) curves computed on both datasets. The simulations were repeated 30 times and their results were averaged to obtain a more accurate evaluation. Both methods show a high true positive rate against low false positive rate, although *ncPred* is clearly able to achieve better results. This is also shown in Table 2, where we can see a significant increase in the average area under the ROC curve (AUC). Such a significance is further proved by the results shown in Table 3. By applying the Friedman rank sum test, we determined that the performance improvement achieved by our algorithm is statistically significant (i.e., the *p*-value is close to zero on both datasets).

**Table 3:** **Friedman rank sum test applied to establish the statistical significance in the performance improvement of ncPred compared to Yang et al. (2014)**.

Dataset	Friedman *χ*^2^	*p*-Value
Chen et al. (2013)	1026.315	<2.2 × 10^−16^
Helwak et al. (2013)	6537.915	<2.2 × 10^−16^

**Table 4:** **Optimal values of *λ*_1_ and *λ*_2_ parameters for the datasets used in our experiments**.

Dataset	*λ*_1_	*λ*_2_
Chen et al. (2013)	0.5	1
Helwak et al. (2013)	0.2	0.2

Regarding the parameters *λ*_1_ and *λ*_2_, we performed a comprehensive analysis to establish the relationship between them and the prediction quality. In the supporting materials, we report the results of such analysis. The results indicate that there is no specific law, which governs their behavior. The peculiar characteristics of each dataset greatly affect the performances and, consequently, the parameters. It is, therefore, necessary to perform an *a priori* analysis in order to determine, which values give the best results. In our experiments, we used such an analysis to determine the best parameters in terms of precision and recall enhancement (see Table 4 for details on their values). By looking at the characteristics of our data sets, the values obtained from such an analysis allowed us to suppose that the two parameters are close to zero in Helwak et al. (2013) dataset because of the greater density. This implies that to maintain high quality predictions it is necessary to reduce their number to avoid the introduction of noise. On the other hand, the Chen et al. (2013) dataset has a lower density. This allows us to produce a higher number of predictions before they start losing quality. Therefore, this explains the lambda values closer to one. It is important to point out that in order to determine the best parameters an analysis was performed considering only precision and recall enhancement, since they are closely related to the biological significance of the predictions. In this context, we report in Table 2 only precision and recall enhancement and the AUC, ignoring the other metrics, which are available in the supporting materials.

Finally, assuming that the number of targets dominates the ncRNA one, we can state that the computational complexity of our method is *O*(*m*^2^*p*). However, it is quite straightforward to implement parallelization and optimization techniques to make the computation faster.

### 3.1 Case studies

The analysis of the predictions for each non-coding showed that *ncPred* is able to find exactly the same predictions provided by Yang et al. (2014). The main difference between the two algorithms lies in the different scores given to each prediction. As highlighted in the previous section, ncPred is clearly able to provide more substantially accurate predictions.

To further demonstrate the ability of our method, we reviewed in detail the results of five diseases (i.e., Alzheimer’s Disease, Myocardial Infarction, Pancreatic Cancer, Parkinson’s disease, and Gastric Cancer) as case studies. The top 10 predicted genes for each case are listed in Table 5. Table 5 also shows the rank obtained by applying on our dataset, the Yang et al. (2014) method. In this context, the two datasets were taken together in order to start from a wider knowledge base.

**Table 5:** **List of top 10 predictions computed by ncPred and their rank obtained with Yang et al. (2014) for five case studies (Alzheimer’s Disease, Myocardial Infarction, Pancreatic Cancer, Parkinson’s Disease, and Gastric Cancer)**.

ncRNA	ncPred rank	Yang et al. (2014) rank	ncRNA	ncPred rank	Yang et al. (2014) rank
**ALZHEIMER’S DISEASE**					
PVT1	1	3	B2 SINE RNA	6	28
MEG3	2	19	TP53TG1	7	22
TUG1	3	32	WRAP53	8	23
lincRNA-p21	4	21	Kcnq1ot1	9	48
CDKN2B-AS1	5	20	Evf2	10	35
**MYOCARDIAL INFARCTION**					
H19	1	43	Kcnq1ot1	6	23
SRA1	2	24	PVT1	7	47
TUG1	3	26	CDKN2B-AS1	8	25
7SL	4	29	B2 SINE RNA	9	17
BDNF-AS1	5	34	Airn	10	18
**PANCREATIC CANCER**					
HOTAIR	1	16	PCAT1	6	40
LINC00312	2	15	ncRNACCND1	7	9
Kcnq1ot1	3	25	Six3OS	8	45
Xist	4	43	Airn	9	14
TERRA	5	10	RepA	10	47
**PARKINSON’S DISEASE**					
PVT1	1	11	LINC00312	6	24
MEG3	2	16	TP53TG1	7	20
TUG1	3	26	WRAP53	8	21
BACE1-AS	4	23	CDKN2B-AS1	9	27
lincRNA-p21	5	19	B2 SINE RNA	10	40
**GASTRIC CANCER**					
PTENP1	1	38	Evf2	6	60
LINC00312	2	15	Airn	7	13
Xist	3	1	TERRA	8	18
PCAT1	4	29	B2 SINE RNA	9	40
Six3OS	5	39	RepA	10	37

#### 3.1.1 Alzheimer’s disease

Alzheimer’s disease (AD) is one of the most common forms of dementia (Hebert et al., 2003). Recent studies indicate that it affects approximately 0.40% of the world population (Brookmeyer et al., 2007). The disease is, at present, untreatable, and it is characterized by a progressive loss of mnemonic, cognitive, and intellectual capacity, which ultimately leads to the death of the patient. Among the first 10 ncRNAs, we find *PVT1* a lncRNA, which regulates the transcription of *MYC* on the long distance (Carramusa et al., 2007). In Jiang et al. (2013), *MYC* has been characterized as the source of the main pathway substantially active in AD, thus having an important role in disease progression. Such a discovery confirms that *PVT1* could play a key role in the progress of AD. We have also identified the lncRNA *MEG3* that activates *TP53* and improves its binding affinity to target gene promoter (Liao et al., 2011). *TP53* was identified in Tan et al. (2012) as potential biomarker for AD. Therefore, further analysis to confirm *MEG3* role in AD are needed.

#### 3.1.2 Myocardial Infarction

Myocardial infarction (MI) is a heart condition that occurs when the proper flow of blood to a part of the heart stops, and the heart muscle is damaged due to lack of sufficient oxygen. Genome-wide association studies have identified 27 epigenetic factors that are associated with an increased risk of MI (Feero et al., 2011). For example, the genomic locus 9p21 has one of the strongest associations with the pathology (Feero et al., 2011). The majority of such factors have been identified in regions implicated in other heart diseases (Feero et al., 2011). Among our predictions, we identified the lncRNA *SRA1* that Friedrichs et al. (2009) found crucial in cardiomyopathies. This leads us to assume a possible link with MI. In the top 10 predictions we also found the lncRNA *7SL*, which, by hybridizing to the *reverse-Alu-element-containing 3′UTR of MnSOD* gene, represses its expression (Lipovich et al., 2010). Overexpression of *MnSOD* has been identified as a possible protection against MI in transgenic mice (Chen et al., 1998). This could be a cue for further investigations to understand the role such a lncRNA.

#### 3.1.3 Pancreatic cancer

Pancreatic cancer is an aggressive disease whose 5-year survival rate is extremely low (Amundadottir et al., 2009). The analysis of the predictions obtained by our algorithm has provided the association with lncRNA *HOTAIR*, whose overexpression has been associated with a poor prognosis in pancreatic cancer, as well as show a pro-oncogenic activity (Kim et al., 2012). A further lncRNA is *Airn*. The deletion of its promoter in paternal allele results in aberrant activation of *IGF2R* (Nagano and Fraser, 2009), whose polymorphisms are associated with an increased risk of pancreatic cancer (Dong et al., 2012).

#### 3.1.4 Parkinson’s disease

Parkinson’s disease (PD) is a degenerative disorder of the central nervous system. The main cause of the disease is the death of dopamine-generating cells in the substantia nigra. The cause of this death is still unknown, nevertheless, the process of aging and metabolic stress are its common triggers (Parlato and Liss, 2014). It is interesting to note that the response to stress conditions and mechanisms for quality control are compromised in patients with PD. The reduction in the transcription of rRNA (ribosomal ribonucleic acid) is an important strategy to maintain cellular homeostasis under stress. An altered transcription is associated with neurodegenerative disorders. There are many triggers for nucleolar stress, but they seem to depend on the extitp53 protein (Parlato and Liss, 2014). Our algorithm is able to identify two probable lncRNA associated with this function: *PVT1*, also associated with AD, whose gene locus is a target of *p53* (Barsotti et al., 2012), and *MEG3* that promotes the expression of *Tp53* and increases the binding affinity to the promoters of its target (Liao et al., 2011).

#### 3.1.5 Gastric cancer

Gastric cancer is a disease typically characterized by an overall 5 years survival rate lower than 10%, mainly due to the plurality of common symptoms that lead to treatments only in advanced disease stages (Orditura et al., 2014). Among our predictions, we find the lncRNA *Xist*. In Weakley et al. (2011), it was identified as differentially expressed in stomach preneoplastic cells, which could be a symptom of gastric cancer. Another factor could be the lncRNA *Evf2*, which is a direct putative positive regulator of transcription factor *Dlx-2* (Lipovich et al., 2010). Increased expression of *Dlx-2* was correlated with more advanced stages of the disease (Tang et al., 2013).

## 4 Discussion

In this paper, we propose *ncPred* to predict novel associations between ncRNAs and diseases. The aim is to compute ncRNA-disease association’s prediction starting from a tripartite network. Such a network integrates information on ncRNAs, targeting (i.e., those genes, microRNAs, proteins whose activity is affected by non-coding RNA), and their associations with diseases in order to improve prediction quality and accuracy.

Our experimental analysis shows that our approach predicts more biologically significant associations with respect to Yang et al. (2014). This assertion is confirmed by the results obtained in terms of recall, which as described above measures biological quality of results. The use of Friedman rank sum test also showed that the difference between our predictions and those of Yang et al. (2014) is not random but due to a better interpretation of available information. The results showed that our method could provide interesting suggestions in the study of the implications between ncRNA and pathologies. However, as stated in the introduction, the method can only help to make such a search more targeted and less expensive, offering a ranking of associations from more probable to less probable. Determine whether those associations are useful still remains within the competence area of bio-physicians that can provide conclusive evidence by identifying suitable patients and documenting such cases.

Despite what stated earlier, our method still has some limitations that should be taken into account. Firstly, ncRNA-target associations are still too small in number. It may be necessary to resort to additional targeting prediction techniques so as to expand original knowledge base. Secondly, the methodology does not use the biological information accompanying each association (e.g., type of ncRNA-target interaction, conditions in which the target-disease association was detected, tissues in which associations have significance). For this reason, it may be useful to further expand the methodology by using such additional information, which could make the methodology more reliable in terms of significant predictions.

## Conflict of Interest Statement

The authors declare that the research was conducted in the absence of any commercial or financial relationships that could be construed as a potential conflict of interest.

## Supplementary Material

In the Supplementary Material (Data Sheet 1.pdf) we report the ncPred parameter tuning further details concerning the comparison with Yang et al. (2014). The Supplementary Material for this article can be found online at http://www.frontiersin.org/Journal/10.3389/fbioe.2014.00071/abstract
