# Supplementary material for: ncPred: ncRNA-Disease Association Prediction through Tripartite Network-Based Inference
Source: Front Bioeng Biotechnol. 2014 Dec 12;2:71. doi: 10.3389/fbioe.2014.00071 (PMC4264506; doi:10.3389/fbioe.2014.00071)
Supplement: Supplementary file 1 [file Data_Sheet_1.PDF]

Frontiers in Bioengineering

Research Article
18 November 2014

Supplementary Material: ncPred: ncRNA-Disease association prediction through tripartite network based inference

Salvatore Alaimo [1], **Rosalba Giugno** [2,*,†], **and Alfredo Pulvirenti** [2,*,†]

[1] *Department of Mathematics and Computer Science, University of Catania, Catania, Italy*
[2] *Department of Clinical and Molecular Biomedicine, University of Catania, Catania, Italy*

Correspondence*:
Alfredo Pulvirenti
Department of Clinical and Molecular Biomedicine, University of Catania, Viale A. Doria 6, Catania, Italy, apulvirenti@dmi.unict.it

Rosalba Giugno
Department of Clinical and Molecular Biomedicine, University of Catania, Viale A. Doria 6, Catania, Italy

Bioinformatics of Non-Coding RNAs with Applications to Biomedicine: Recent Advances and Open Challenges

1 INTRODUCTION

Here we provide a comprehensive experimental analysis of **ncPred**. Since **ncPred** relies on the usage of two parameters λ_1 and λ_2 we aim to investigate its behavior by varying them. Next we will show the detailed comparison of **ncPred** against **Yang et al.** (2014).

2 SUPPLEMENTARY TABLES AND FIGURES

In this section we first report the analysis of the algorithm by varying the two parameters λ_1 and λ_2 . The ranges of values for both parameters are between 0 and 1, with a step of 0.1. In Figures 3 and 4, we show the results (by means of an heatmap) of our study on the dependence of the four metrics on such parameters. In the x-axis we report the value of λ_1 , and in the y-axis the value of the parameter λ_2 . The bar to the left of each graph associates each color with the value assumed by a particular metric. Results show that we cannot determine a law that regulates the behavior of the metrics based on the values of these parameters. Therefore we concluded that they depend heavily on the specific characteristics of each dataset, and is, therefore, required a priori analysis in order to select the best ones.

Next, we report the detailed results of the comparisons among **ncPred** and **Yang et al.** (2014). Tables 1 and 2 provide the results of the four metrics (Recovery, Precision and Recall Enhancement, Personalization, Surprisal) and the average under the ROC curves (AUC).

† Equal contributor

Finally in figure 1 and 2 we show a rendering of the two tripartite networks used as datasets. In these images we reported each type of nodes using different colors: ncRNAs in blue, targets in orange, and diseases in red.

Supplementary Table 1. The four metrics (Recovery, Precision and Recall Enhancement, Personalization, Surprisal) and the average area under ROC curve (AUC) computed for **ncPred** and **Yang et al.** (2014) (in boldface are highlighted best results) applied to **Chen et al.** (2013) dataset.

Algorithm	r	$e_P\,(20)$	$e_R\,(20)$	$h\,(20)$	$I\,(20)$	AUC
**ncPred**	**0.5299**	**12.3290**	**1.6636**	0.3875	5.2885	**0.7566** $\pm$ **0.0218**
**Yang et al.** (2014)	0.6194	5.5113	0.7297	**0.5562**	**5.8057**	0.6217 $\pm$ 0.0178

Supplementary Table 2. The four metrics (Recovery, Precision and Recall Enhancement, Personalization, Surprisal) and the average area under ROC curve (AUC) computed for **ncPred** and **Yang et al.** (2014) (in boldface are highlighted best results) applied to **Helwak et al.** (2013) dataset.

Algorithm	r	$e_P\,(20)$	$e_R\,(20)$	$h\,(20)$	$I\,(20)$	AUC
**ncPred**	**0.5650**	**5.8197**	**5.6572**	0.5475	1.0717	**0.7669** $\pm$ **0.0093**
**Yang et al.** (2014)	0.6520	1.8654	1.6509	**0.7317**	**2.7767**	0.7069 $\pm$ 0.0084

REFERENCES

Chen, G., Wang, Z., Wang, D., Qiu, C., Liu, M., Chen, X., et al. (2013), Lncrnadisease: a database for long-non-coding rna-associated diseases, *Nucleic Acids Research*, 41, D1, D983–D986, doi:10.1093/nar/gks1099

Helwak, A., Kudla, G., Dudnakova, T., and Tollervey, D. (2013), Mapping the human mirna interactome by clash reveals frequent noncanonical binding, *Cell*, 153, 3, 654–665

Yang, X., Gao, L., Guo, X., Shi, X., Wu, H., Song, F., et al. (2014), A network based method for analysis of lncrna-disease associations and prediction of lncrnas implicated in diseases, *PLoS ONE*, 9, 1, e87797, doi:10.1371/journal.pone.0087797

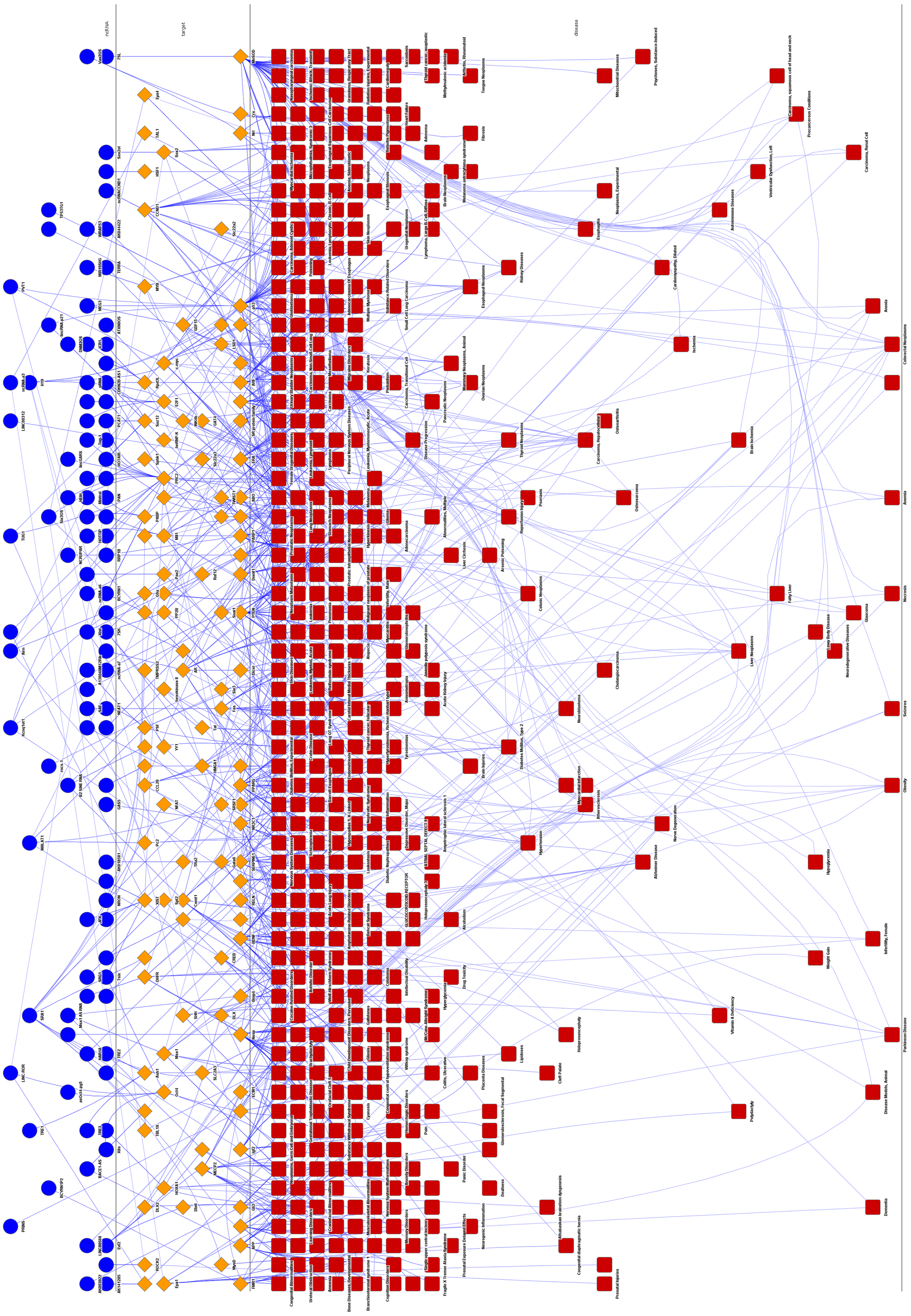

Supplementary Figure 1. Rendering of **Chen et al.** (2013) tripartite network used for the prediction of ncRNA-disease associations. In the image we highlight nodes associated with ncRNAs in blue, targets in orange, diseases in red.

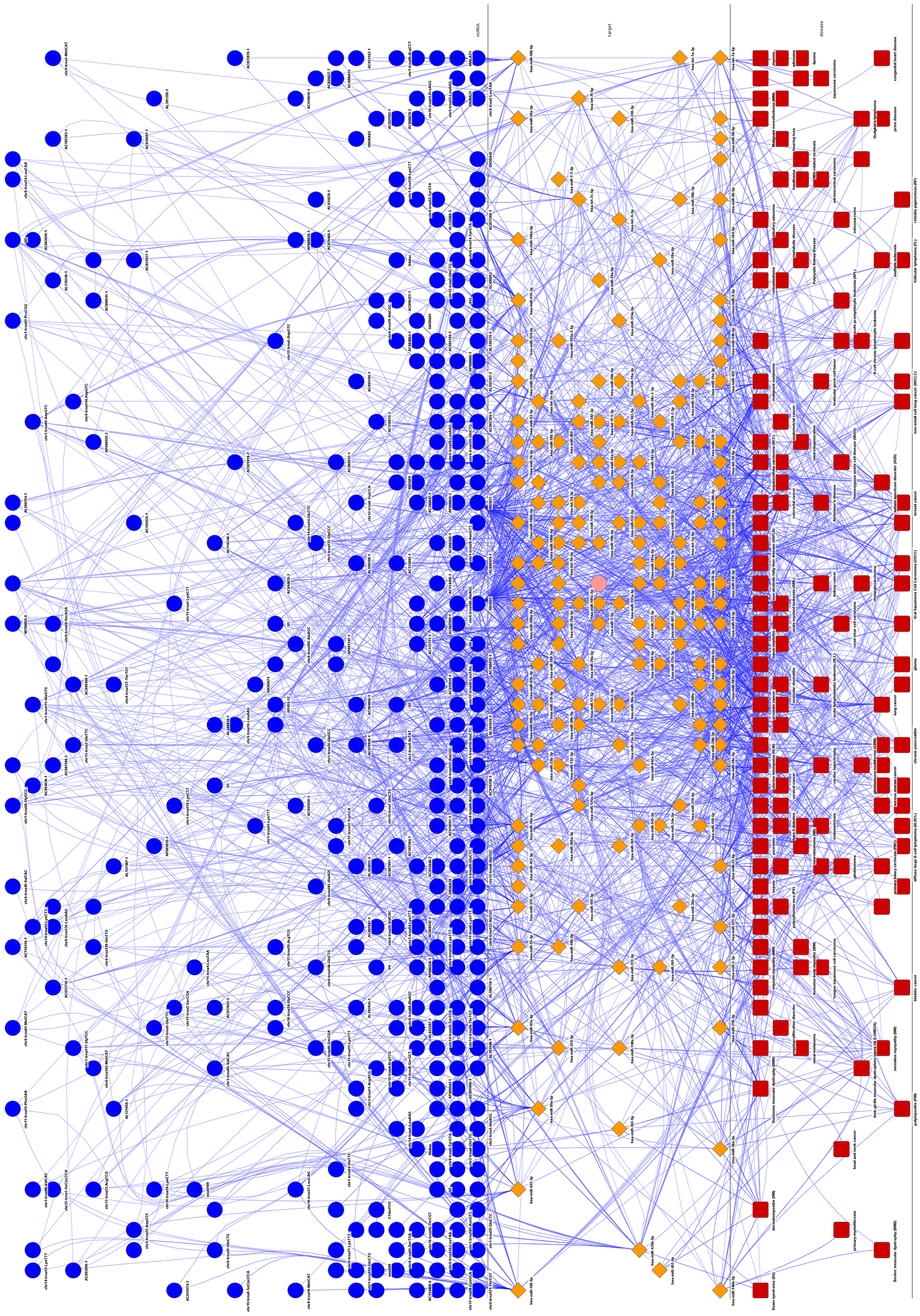

Supplementary Figure 2. Rendering of **Helwak et al.** (2013) tripartite network used for the prediction of ncRNA-disease associations. In the image we highlight nodes associated with ncRNAs in blue, targets in orange, diseases in red.

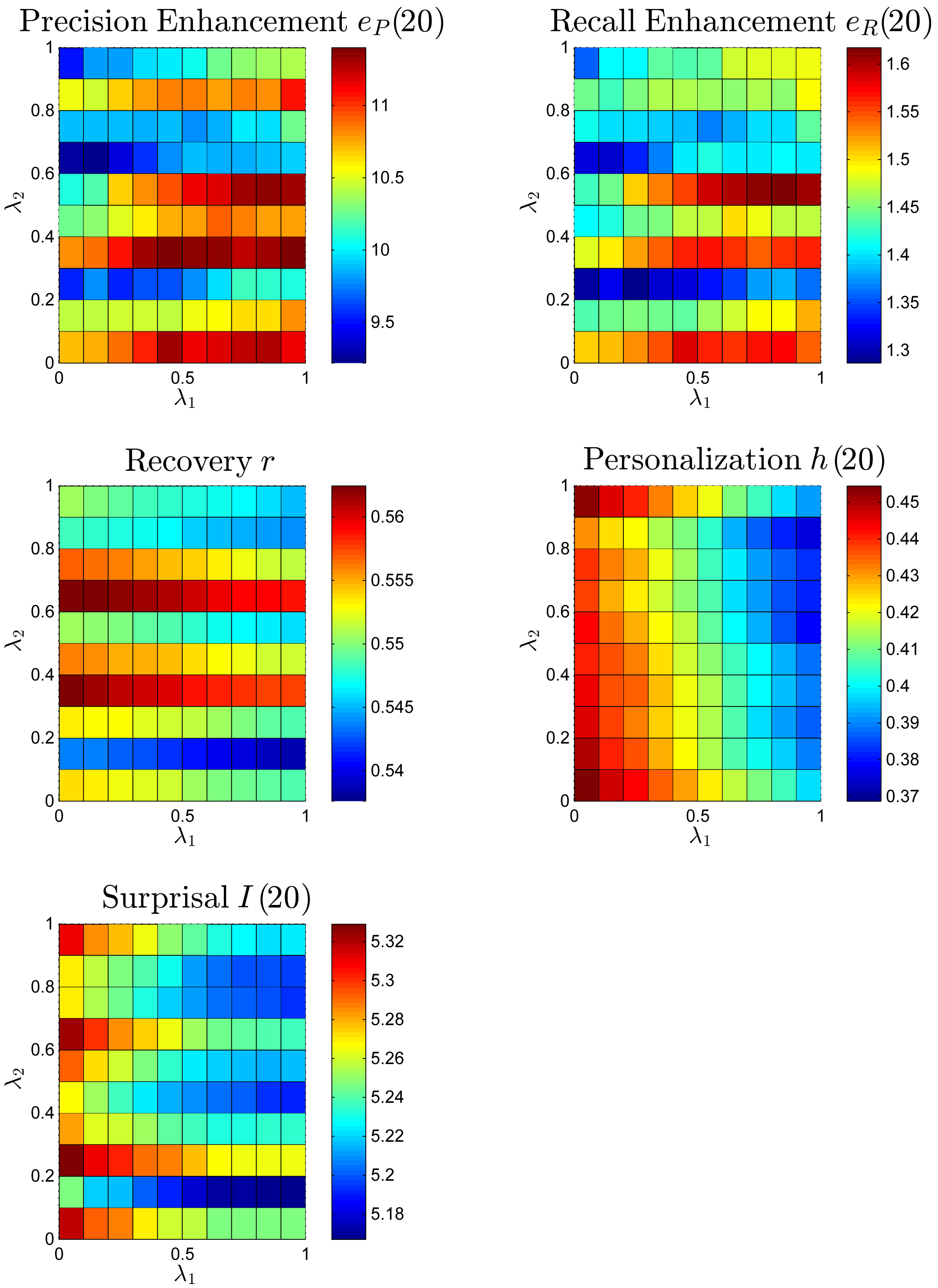

Supplementary Figure 3. Dependence of the metrics on **ncPred** λ_1 and λ_2 parameters for the **Chen et al.** (2013) dataset.

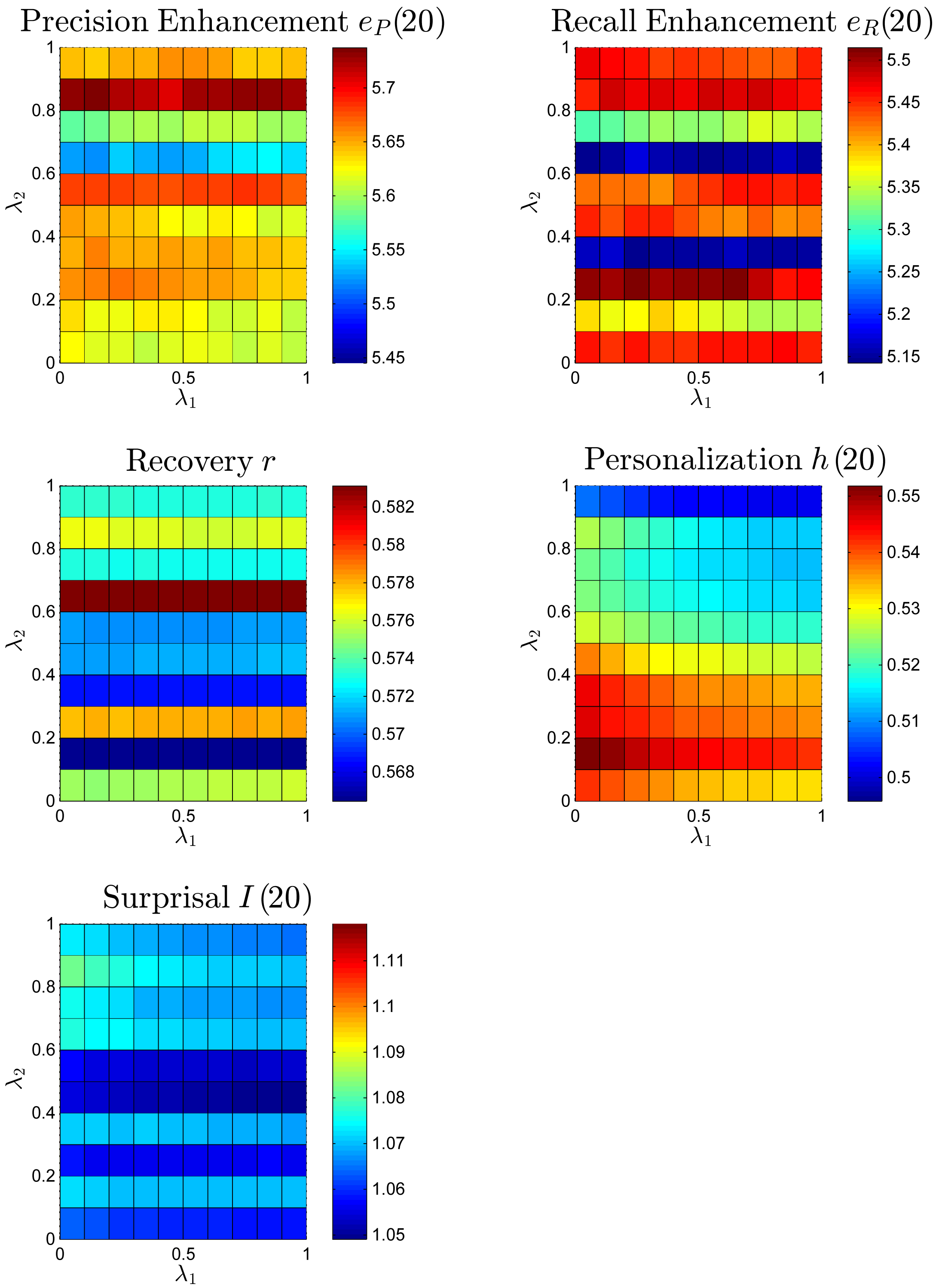

Supplementary Figure 4. Dependence of the metrics on **ncPred** λ_1 and λ_2 parameters for the **Helwak et al.** (2013) dataset.
